# *How deep is your thought?* The relations between intolerance of uncertainty, worry and weight and shape concerns in adolescent girls with anorexia nervosa

**DOI:** 10.1186/s40337-021-00523-4

**Published:** 2021-12-20

**Authors:** Jojanneke M. Bijsterbosch, Anouk Keizer, Paul A. Boelen, Femke van den Brink, Unna N. Danner, Lot C. Sternheim

**Affiliations:** 1 Department of Clinical Psychology, Utrecht University, PO Box 80140, 3508 TC Utrecht, The Netherlands; 2 Department of Experimental Psychology, Utrecht University, Utrecht, The Netherlands; 3 ARQ National Psychotrauma Centre, Diemen, The Netherlands; 4 ARQ Centrum’45, Diemen, The Netherlands; 5 Rintveld Center for Eating Disorders, Altrecht, Zeist, The Netherlands

**Keywords:** Adolescent girls, Anorexia nervosa, Weight and shape concerns, Prospective intolerance of uncertainty, Inhibitory intolerance of uncertainty, Worry

## Abstract

**Background:**

Inherent to anorexia nervosa are repetitive thoughts about weight and shape. Growing research suggests the relevance of intolerance of uncertainty and worry in maintaining these types of repetitive thoughts. The relation between these cognitive processes and weight and shape concerns in adolescent girls with anorexia nervosa is understudied. This study investigated associations between prospective (desire for predictability) and inhibitory (uncertainty paralysis) intolerance of uncertainty, and weight and shape concerns and the mediating role of worry in these associations.

**Methods:**

In a cross-sectional study, 93 adolescent girls with anorexia nervosa completed questionnaires measuring the variables of interest. A mediation model with worry as a mediator between inhibitory and prospective intolerance of uncertainty and weight and shape concerns was tested.

**Results:**

A total and direct effect of inhibitory intolerance of uncertainty on weight and shape concerns was found. Worry did not mediate this relation.

**Conclusions:**

These results confirm the importance of inhibitory intolerance of uncertainty in adolescent girls with anorexia nervosa, more specifically to weight and shape concerns. This group may benefit from intervention strategies targeting intolerance of uncertainty. General worry seems less relevant to weight and shape concerns in adolescent girls with anorexia nervosa.

**Plain English summary:**

Adolescent girls with anorexia nervosa often experience repetitive thoughts about weight and shape. Growing research suggests the relevance of intolerance of uncertainty and worry in maintaining these types of repetitive thoughts. Intolerance of uncertainty is defined as the incapacity to tolerate uncertainty and is often divided into two components; prospective intolerance of uncertainty (desire for predictability) and inhibitory intolerance of uncertainty (uncertainty paralysis). The relation between intolerance of uncertainty, worry and weight and shape concerns in adolescent girls with anorexia nervosa is understudied. This study aims to investigate study relations between prospective and inhibitory intolerance of uncertainty, worry, and weight and shape concerns. A total of 93 adolescent girls with anorexia nervosa completed three questionnaires, measuring prospective and inhibitory intolerance of uncertainty worry, and weight and shape concerns, respectively. The results of this study confirmed the importance of inhibitory intolerance of uncertainty in adolescent girls with anorexia nervosa, more specifically to weight and shape concerns. This group may benefit from intervention strategies targeting intolerance of uncertainty. General worry seems less relevant to weight and shape concerns in adolescent girls.

## Introduction

Key diagnostic features of anorexia nervosa (AN) include the disturbance in the way in which one’s body weight and shape is experienced and undue influence of body weight and shape on self-evaluation [1]. The typical onset of AN is during adolescence or young adulthood [13] with a peak at 15 to 17 years [68]. Puberty coincides with rapid changes in body size and shape, which then must be integrated within one’s body image [58]. These rapid changes can result in increases in weight and shape concerns [11, 26, 47, 49] which, in turn, are important risk factors and maintaining factors for AN [26, 37, 69]. Weight and shape concerns entail the subjective negative appraisal of one’s body and the overvalued ideals about the personal implications of weight and shape [29]. Examining the mechanisms underlying weight and shape concerns in adolescent girls with AN might improve our understanding of the extreme body image disturbances that are frequently observed in AN. Moreover, individuals who are recovered from AN still show a partially disturbed body image [24]. A better understanding of body image disturbances in adolescent girls with AN may help to identify predictors of AN symptomology at an earlier stage of the disorder. This knowledge may translate to more suited and tailor made interventions for AN, specifically targeting IU and worry. This study sought to examine the role of worry and intolerance of uncertainty in weight and shape concerns in adolescent girls with AN.

By definition, AN involves repeatedly thinking about weight and shape, which can take up large parts of the day [61]. Indeed, literature is starting to show that these types of repetitive thoughts are part of the anxious, rigid and obsessional phenotype associated with AN [46]. Preliminary evidence suggests the relevance of a number of core cognitive anxiety-related processes to AN [66], such as intolerance of uncertainty (IU) and worry [63, 65].

IU has been defined as an individual’s dispositional incapacity to endure the aversive response triggered by the perceived absence of salient, key or sufficient information, and is sustained by the associated perception of uncertainty [14]. A number of studies reported significantly higher degrees of IU in AN compared to those with other types of eating disorders or healthy controls [10, 39, 64], however research examining IU in children and adolescent girls is scarce [43]. Two studies identified elevated levels of IU in adolescent girls with AN [28, 43]. Individuals with high levels of IU perceive uncertainty as threatening [14, 15]. In those with AN, uncertainty related to weight gain and changes in shape could easily turn into an unacceptable threat and, as a result, hinder treatment focused on weight gain. Moreover, as changes in weight and shape are one of the most dominant characteristics of adolescence, the biologically driven development of body weight and shape might become even more intolerable to adolescent girls with high IU. Preliminary evidence confirms that IU is associated with weight and shape concerns in adult women with AN [10, 28] and in non-clinical women [3]. However, studies in adolescent girls with AN have not yet been conducted.

Research has demonstrated that IU is made up of two separate but related factors: prospective IU and inhibitory IU (e.g., [4, 16, 34, 50]). Prospective IU refers to a desire for predictability that is driven by a sense of uneasiness with uncertainty [34]. It represents the negative cognitive appraisals of possible future uncertain outcomes. Inhibitory IU refers to the inhibition of action or experiences as a result of apprehension of uncertainty. Individuals high in inhibitory IU freeze up in the face of uncertainty and engage in avoidance strategies, including cognitive avoidance strategies like worry [8]. As such, inhibitory IU might be slowing down or even hindering progress in therapy when trying to use experimental or other behavioral treatment strategies to work through and diminish weight and shape concerns. Prior research has identified inhibitory IU as the most toxic component of the two as can be seen from its strong association with psychopathological symptoms and cognitive vulnerabilities such as worry in adults [4, 34], as well as in adolescents [5].

The main feature of worry is the predominance of negative-type, repetitive, and preoccupied thought about possible threatening future events [7, 75]. The predictive value of IU to worry has been firmly established across many psychiatric disorders such as generalized anxiety disorder [42], obsessive compulsive disorder [51], social anxiety [5] and eating disorders (ED; [10]); negative beliefs about uncertainty may lead to difficulty dealing with uncertainty which, in turn, may lead to excessive worry [22].

Preliminary evidence shows that worry levels are significantly higher in adults with ED compared to those without ED, and particular in people with AN [38, 63, 65]. Moreover, higher levels of worry are associated with more severe ED symptomatology, including weight and shape concerns [38, 57, 63, 66]. In addition, a study by Sternheim et al. [65] confirmed that weight and shape concerns are indeed incorporated in worries reported by adults with AN. As of yet, research has not studied this link in an adolescent population. This is relevant, because from a developmental perspective, worry is known to increase and change with age [22, 45].

Individuals affected by intolerance of negative emotions tend to use worry to avoid experiencing negative emotions [60, 77]. As worrying might serve as a distraction [73] from more distressing thought content (e.g., relationships or social problems), it is plausible that adolescent girls with AN use worry about weight and shape concerns to diminish the emotions connected with these concerns for this purpose. Moreover, a recent study indeed suggested that from a behavioral learning perspective, children prone to anxious feelings and worries may be more likely to develop concerns related to weight and body image as adolescents and may then seek out behaviors that offer mitigate these concerns [57, 59]. A handful of studies in clinical and non-clinical adult populations show that worry is indeed related to body dissatisfaction [40, 56, 57, 65] and a longitudinal study found that child worries reported by parents at age 10 were predictive of a later onset of AN at age 14 [59].

In sum, inherent to the pubertal transformation of the female body is uncertainty about both changes during this process and the grown body. Adolescent girls who have difficulties tolerating the uncertainty related to this bodily transformation process, may be expected to experience an increase in weight and shape concerns. Moreover, one could speculate that worry may impact the relation between IU and weight and shape concerns, as worry is a cognitive strategy that functions to reduce these anxiety provoking feelings of uncertainty [60, 77]. Considering the broader literature on IU and worry in emotional disorders, it is conceivable that IU precedes worry and not vice versa (e.g., [41, 45, 78]). This study aims to test associations between IU and weight and shape concerns through an indirect path via worry.

**Fig. 1:**
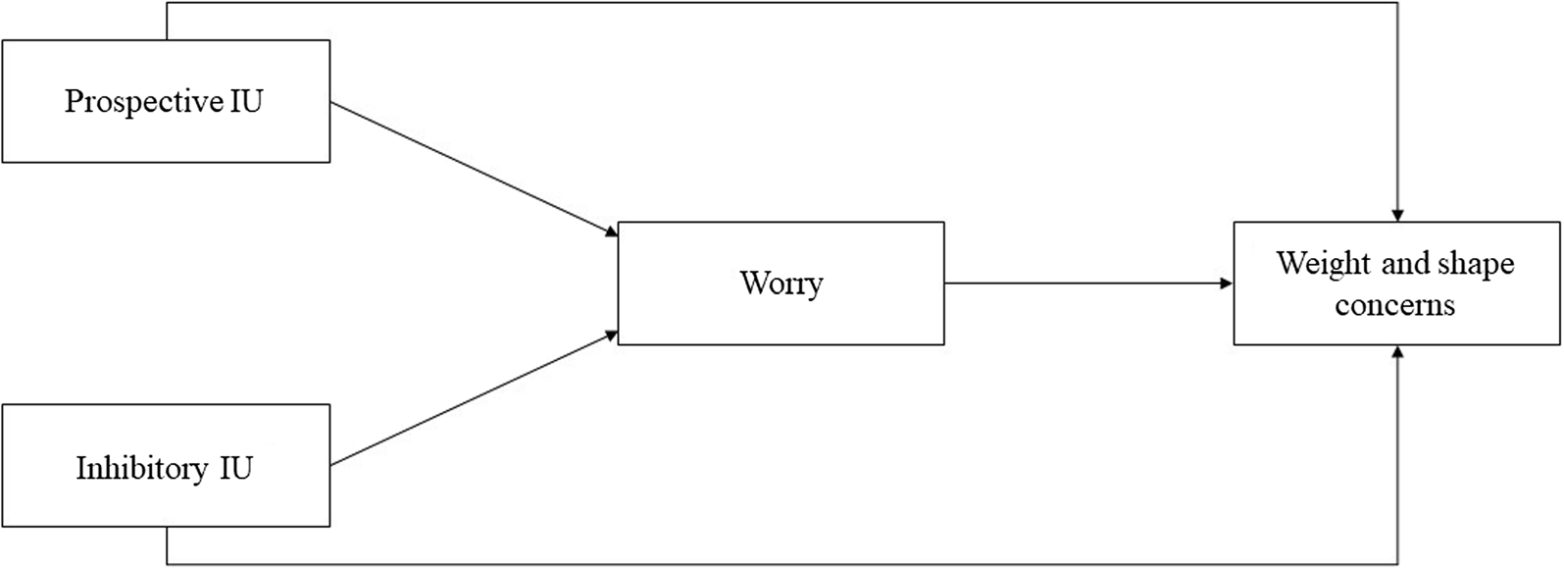
Schematic summary of the hypothesized associations between prospective and inhibitory IU with weight and shape concerns and the mediating role of worry. *Note* Inhibitory IU = Inhibitory intolerance of uncertainty; Prospective IU = prospective intolerance of uncertainty

Based on previous findings (e.g., [28]), it was expected that higher levels of IU were related to higher levels of weight and shape concerns. Additionally, a positive indirect association between IU and weight and shape concerns through worry was expected. Previous studies identified inhibitory IU as the most toxic component in the context of psychopathological symptoms and worry, relative to prospective IU (e.g., [4, 34]). Hence, it was expected that the proposed mediation model was particularly salient for inhibitory IU, compared to prospective IU. The hypothesized associations between prospective and inhibitory IU with weight and shape concerns and the mediating role of worry are schematically depicted in Fig. 1.

## Method

### Participants and procedure

This study is part of a larger study into the geno- and phenotypes in people with EDs, conducted by a specialized treatment facility in the Netherlands. Data for the current study were extracted from this larger database. Participants were included into the current study when meeting all the following criteria: age 12 to 18 years, female gender, a DSM-5 [1] diagnosis of AN restricting (ANR) subtype or binge eating/purging (ANBP) subtype, and no missing data in the study’s variables.

Participants were recruited at the time of intake at the treatment facility, and participation took place within this same period, at the beginning of treatment. Before participating, participants were informed about the procedure and were given the opportunity to ask questions about the study. Participants who then signed the informed consent form were enrolled in the study. The EDE interview was administered by trained advanced clinical psychology students at the treatment facility. The EDE interview is part of the intake procedure and is scheduled to take approximately 60 min. All other measures were completed on a computer at the treatment facility, which were programmed using Inquisit software (version 4; Millisecond, 2016). Participants were told to read the instructions of each questionnaire carefully. Completing the entire test battery took approximately 45–60 min. The researcher was nearby, in case there were any questions. Afterwards, participants were debriefed and given the opportunity to indicate whether or not they wanted to be informed about the results of the study. The research protocol was authorized by the Committee Scientific Research of Altrecht Mental Health Institute and the Medical Ethical Committee of the University Medical Center Utrecht.

### Measures

#### Intolerance of uncertainty

The Intolerance of Uncertainty Scale (IUS-12; [16]) measures one’s IU as expressed in several domains, including emotion, cognition and behavior. It has two subscales, tapping prospective IU and inhibitory IU, respectively. Respondents rate the degree to which each of 12 items apply to them on 5-point Likert scale ranging from 1 (*not at all characteristic of me*) to 5 (*entirely characteristic of me*). An example of an item of the prospective IU subscale is “It frustrates me not having all the information I need.” An example of the inhibitory IU subscale is “When it is time to act, uncertainty paralyses me”. Subscale scores were calculated by summing up the respective items, with higher scores indicating higher levels of IU. The Dutch version of the IUS-12 has good psychometric properties [33]. In the current study, the IUS-12 total had a good internal consistency (α = 0.89), the internal consistency of the prospective IU subscale was considered good (α = 0.86) and that of the inhibitory IU subscale (α = 0.75) was considered acceptable.

#### Worry

The Penn State Worry Questionnaire (PSWQ; [52]) contains 16 items that assess pathological worry on a 5-point Likert scale ranging from 1 (*not at all typical*) to 5 (*very typical*). An example item is “My worries overwhelm me.” Total scores were calculated by summing all the item scores (after reversing some of them), resulting in a possible range of 16 to 80. Higher scores indicate higher levels of pathological worry. The Dutch PSWQ has very good psychometric properties [38]. The PSWQ had good internal consistency in the current study (α = 0.84).

#### Weight and shape concerns

The Dutch version of the Eating Disorder Examination (EDE, 12th edition; [25, 36]) was conducted and is widely regarded as the “Gold Standard’ to measure of eating disorder psychopathology and behaviors. It is an investigator-based clinical interview that provides a comprehensive assessment of the frequency and the severity of key behavioral and psychological aspects of eating disorders [25]. It focuses on the past 28 days and assesses the main behavioral and attitudinal features of eating disorders. The behavioral features are measured in terms of their frequency and the number of days on which they occurred. For the remaining items, the interviewer rates the participant’s response on a 0–6 scale of severity, with higher scores indicating greater levels of psychopathology. It comprises four subscales: Restraint, Shape Concern, Weight Concern and Eating Concern. For the purpose of this study only the subscales Weight Concern and Shape Concern are used. The mean of the items of these subscales was calculated, considering that factor analysis suggests that the items of both subscales generally load on one factor [74]. The EDE has demonstrated good internal consistency, inter-rater reliability and convergent and discriminant validity for the subscales in samples of adolescents [70]. Wade et al. [74] reported an excellent internal reliability for the weight and shape subscales (α = 0.91).

### Statistical analysis

The statistical analyses were performed using IBM SPSS Statistics Version 26 [35] and PROCESS for SPSS v3.0 [32]. Scores on IUS12 and EDE were normally distributed. Scores on the PSWQ slightly violated the assumption of normality [27] and some outliers were detected. However, the sample of this study was large enough (*N* > 40) to be able to use parametric procedures without causing any major problems, despite PSWQ data not being normally distributed [30]. First of all, bivariate associations between the study variables were analysed using Pearson’s correlation coefficients. Then, a mediation analysis with prospective IU and inhibitory IU as predictors, worry as mediator and weight and shape concerns as criterion variable was conducted. The mediation analysis comprises the following steps ([32]; model 4): first, in order to estimate the unique effects of prospective and inhibitory IU on worry, a multiple regression analysis was performed. Second, a hierarchical regression analysis was run in order to estimate the unique total effects of prospective and inhibitory IU (Step 1) and the unique direct effects of prospective and inhibitory IU as well as worry (Step 2) on weight and shape concerns. Third, the unique indirect effects of prospective and inhibitory IU on weight and shape concerns via worry were determined by means of bootstrap analyses with 5000 bootstrap samples [32]. With regard to the direct and indirect effects, PROCESS model 4 was carried out two times,each time putting one dimension of IU as predictor and the other dimension as control variable. Each time PROCESS was run, the direct and indirect effect of the predictor was estimated. Mathematically, all resulting paths, direct and indirect will be the same as if they had been estimated simultaneously (as in a structural equation modeling program; [32]. As previous research has shown that certain features could impact the clinical presentation of AN (e.g., [2]), age of onset, duration of illness and BMI were entered as control variables as well. Standardized coefficients are reported. To determine the efficacy of the regression model, Cohen *f*^2^ effect sizes were calculated [20].

## Results

### Descriptive statistics

A total of 93 participants (76 ANR and 17 ANBP) participated in this study. Participants had an average age of 15.91 years (*SD* = 1.64) and an average amount of educational years of 10.44 (*SD* = 2.19). The average global EDE score was 3.4 (*SD* = 1.23), which was lower than the global EDE score (4.0; *SD* = 1.5) found in a similar sample [12]. The average BMI was 16.93 (*SD* = 2.17), placing the BMI-for-age at the sixth percentile just within the range of a healthy weight (below the fifth percentile is indicative for underweight [17]; https://www.cdc.gov). The average age of onset of AN was 14.11 (*SD* = 1.67), and the average illness duration was 1.80 years (*SD* = 1.51). Table 1 displays the means, standard deviations and the bivariate correlations between the study’s variables. The levels of prospective and inhibitory IU found in this study were higher in comparison to a nonclinical adolescent population [5]. In addition, the levels of worry obtained in this study were comparable to a nonclinical adolescent female group [53] and clinical adults with AN [57], however, lower than the levels found in adults with AN of other studies [63, 64]. The levels of weight and shape concerns found in this study fell within the range to be expected in a clinical sample of adolescent girls with AN [12]. A moderate correlation was found between prospective IU, inhibitory IU and worry. The correlation between inhibitory IU and weight and shape concerns was considered moderate and the associations prospective IU and weight and shape concerns was weak. Worry did not correlate with weight and shape concerns.

**Table 1:** Means, standard deviations and bivariate correlations of analysis measures (N = 93)

	*M*	*SD*	1	2	3	4
1. Prospective IU	19.58	4.64	–	–	–	–
2. Inhibitory IU	15.94	3.89	0.67**	–	–	–
3. PWSQ	46.12	6.38	0.46**	0.41**	–	–
4. EDE weight and shape concern	3.63	1.38	0.22*	0.45**	0.14	–

EDE = Eating Disorder Examination, IU = intolerance of uncertainty; PSWQ = Penn State Worry Questionnaire

*p < .05; **p < .01

### Total, direct, and indirect effects of prospective and inhibitory IU on weight and shape concerns through worry

**Fig. 2:**
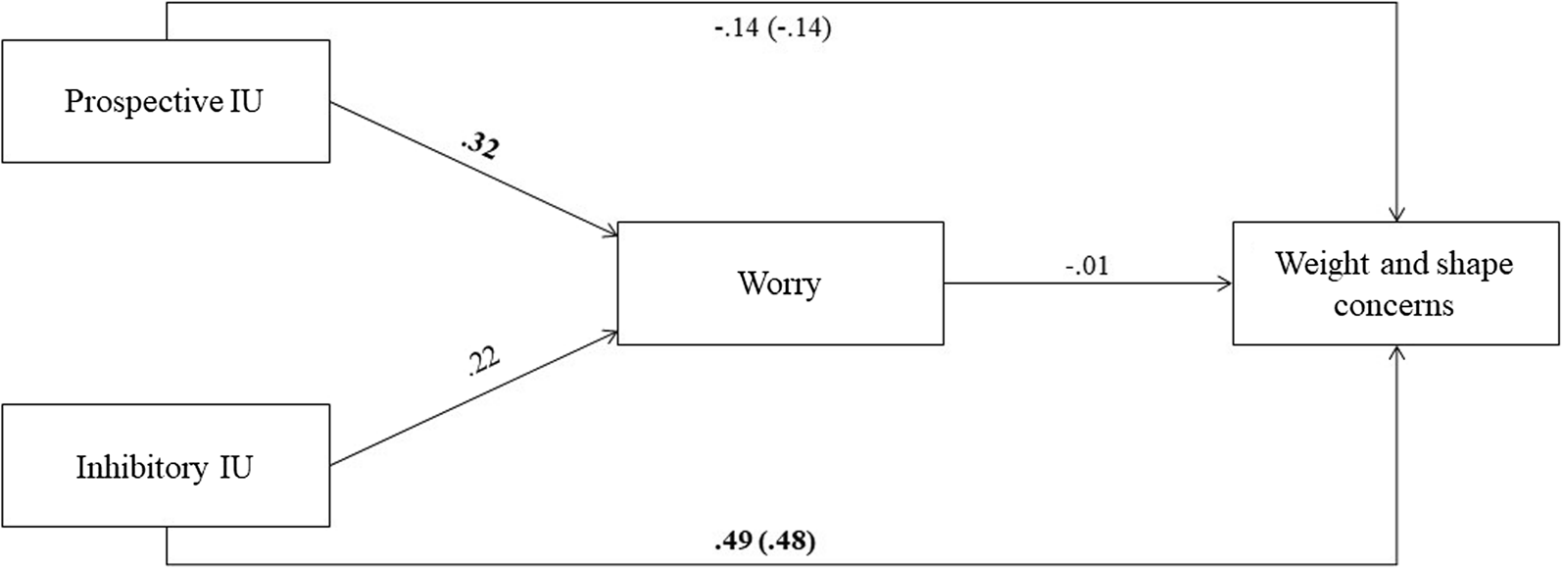
Results of the regression analysis. *Note* Inhibitory IU = Inhibitory intolerance of uncertainty; Prospective IU = prospective intolerance of uncertainty. Coefficients in parentheses represent total effects. Coefficients highlighted in bold are significant of *p* < .01, when controlling for age of onset, duration of illness and BMI

The multiple regression analysis revealed a significant effect of prospective IU and a nonsignificant effect of inhibitory IU (see Fig. 2). A total of 25.4% of the variance in worry could be explained, *F*(5, 87) = 5.910, *p* < 0.001; Cohen’s *f*^2^ = 0.34, which is considered a medium (but near to a large) effect [20].

The hierarchical regression revealed a nonsignificant negative total effect of prospective IU and a significant positive total effect of inhibitory IU in Step 1. In addition, a nonsignificant negative direct effect of prospective IU on weight and shape concerns was revealed and a significant positive effect of inhibitory IU on weight and shape concerns in Step 2 (see Fig. 2). Furthermore, in Step 2, a nonsignificant negative effect of worry on weight and shape concerns was found (see Fig. 2). A total of 27.4% of the variance in weight and shape concerns could be explained *F*(6, 86) = 5.416, *p* < 0.001; Cohen’s *f*^2^ = 0.38, which is considered a large effect [20].

The bootstrap analyses revealed a nonsignificant negative indirect effect of prospective IU,.-0.003, BC 95% confidence interval (CI) [0.04, − 0.09], and a nonsignificant negative indirect effect of inhibitory IU, − 0.002, BC 95% CI [0.03, − 0.05], on weight and shape concerns through worry. Worry thus did not mediate the relationship of both prospective IU and inhibitory IU with weight and shape concerns.

## Discussion

The present study investigated the relationships of prospective IU and inhibitory IU with worry and weight and shape concerns. More specifically, the mediating role of worry in the associations between the IU dimensions and weight and shape concerns was examined. Taken together, our results partly confirmed our hypotheses.

Regarding our IU hypotheses, we expected higher levels of inhibitory IU to be associated with weight and shape concerns, relative to prospective IU. Indeed, only the association of inhibitory IU with weight and shape concerns was found. This study was the first, to the best of our knowledge, to investigate relations between weight and shape concerns and the two components of IU separately in a clinical adolescent AN sample. Our expectations were based on previous clinical studies finding total scores of IU associated with weight and shape concerns in AN [28] and on results from a study by Boelen and Lenferink [4], suggesting that clinically inhibitory IU may be of more importance to more severe ED symptomatology. Indeed results confirm the importance of inhibitory IU to AN, and specifically to weight and shape concerns, which is reflected in the large effect size.

Although the levels of worry were in accordance with the levels of worry found by Sassaroli et al. [57], levels were not as high as found in more recent studies of adults with AN [63]. Worry levels in the present study can be interpreted as moderate,individuals that can be bothered by worries but are just below clinical range for worry [52]. Moderate worry still serves as an adaptive process as it prepares individuals for future threat and increases motivation among other things [21, 62]. However, when moderate worry turns into pathological worry it becomes of clinical concern. Notably, interpreting these results is a relatively complex matter as this is first study investigating worry in adolescent girls with AN which makes it impossible to compare current results to other studies investigating a similar population. Additionally, as excessive worry is often observed in adults with AN, it is suggested that worry levels should always be monitored closely in therapy and targeted when necessary as worry is suggested to be particular relevant for AN development which may contribute to shared risk to anxiety disorders and AN [48, 59].

Interestingly, whilst clinical levels of worry have been detected in adults with AN (e.g., [65]), we failed to establish an association between worry and weight and shape concerns and as a result we did not replicate these findings in the present sample of adolescent girls with AN. One explanation might be related to the contents of worry. As worry becomes increasingly elaborate and abstract during adolescence [72], contents shift from worrying about e.g., the monster under the bed in childhood to more broader psychopathological issues such as the fear of rejection or the fear of being evaluated negatively in puberty [45, 64]. One could speculate that adolescent girls with AN feel overwhelmed by and struggle with managing these changes and that as a possible result, they fill the content of their worries with ED—specific worries which at this stage makes them feel safe and in control. It has been suggested that worry serves the short-term purpose of distraction or relief from more terrifying thoughts (e.g., relationships or social problems), but may lead to weight and shape concerns at a later stage [56]. Indeed, a recent longitudinal study found that child worries reported by parents at age 10 were predictive of a later onset of AN at age 14 and more specifically of body dissatisfaction and weight concerns [59]. As such, worry may still function as a facilitator of positive beliefs about worry (e.g., worrying can reduce uncertainty; [23]) in adolescent girls with AN and is not yet experienced as a salient component of the repetitive thoughts that are bothering many adults with AN during the entire day. To examine this theory, longitudinal studies are required assessing these developmental elements in these relations in patients with AN during their transition from adolescence into adulthood. Future studies should also explore the exact nature of worry but also its different functions to further clarify the complexity of this construct.

Regarding the associations of prospective and inhibitory IU and worry, it was found that only prospective IU was related to worry when controlling for inhibitory IU. Specifically, these higher levels of prospective IU and its association with higher levels of worry do not corroborate with earlier studies in nonclinical adolescents [5] and nonclinical adults [4]. In these studies, it was found that higher levels of inhibitory IU were associated with higher levels of worry, although worry levels were much lower in both studies. Nonetheless, this study replicates findings from other studies that highlight IU as an important contributing factor to worry (e.g., [65]). In addition, differentiating between prospective and inhibitory IU in the association with worry might impact approaches in intervention. Findings from the present study contribute to a growing body of literature positing IU as an important factor that can be linked to the development and maintenance symptoms of AN such as weight and shape concerns [28, 64, 67]. Previous studies have shown that IU is a malleable mechanism and CBT-type interventions for IU have shown success in reducing IU [9]. Furthermore, experimental studies in adult samples have shown that changes in IU lead to corresponding changes in worry [31, 44, 54, 55]. One could argue that adolescent girls with AN may benefit from additional interventions that target IU as well as diminishing levels of IU might help to prevent worry from growing and becoming an integrative part of the repetitive thinking style as observed in adult populations with AN [65].

As of yet, we do not know whether addressing IU will also help to lower levels of weight and shape concerns, possibly contributing to an improved path to recovery. Individuals with AN who experience more severe ED pathology problems such as weight and shape concerns are more likely to have a worse outcome and may be at greater risk of dropping out of treatment [71]. IU might well be part of the mechanism underlying these body image disturbances. Future research should focus on body image related IU and how to target this in interventions that are suitable for adolescent girls with AN. It may be beneficial to address IU among individuals with AN in order to prevent worry from becoming part of the repetitive thinking style and diminish ED symptoms such as weight and shape concerns along the way.

Some limitations need to be acknowledged. Due to the cross-sectional design, the direct of causality in the associations between the constructs could not definitely be determined [76]. Furthermore, whilst a large majority of cross-sectional and treatment studies have identified IU as a predictor of worry rather than vice versa (e.g., [41, 78]), there are a couple of studies that suggest a reciprocal relation, with worry contributing to IU (e.g., [6]). The current dataset only allowed for investigating cross-sectional associations and longitudinal studies may show the developmental patterns of IU and worry in adolescent girls with AN over time. In addition, a longitudinal study that could start in early adolescence and follow up into adulthood would provide a better insight in the development of the transition of nonclinical worry in an adolescent sample into pathological levels of worry as is seen in an adult AN sample (e.g., [63, 65]). Furthermore, future might focus on for instance general worry as well as on positive beliefs about worry and eating disorder specific worry. In doing so, the precise nature and function of worry might be further unraveled and result into an improved understanding of worry. Although the carefully selected PSWQ has been the most widely-used measure of the frequency, intensity, and uncontrollability of worry and it has been employed within both clinical and non-clinical populations [52], it could well be that other instruments would have been more suited to capture the construct of worry within a population of adolescent AN girls with a mean age of almost 16 years old. Using the PSWQ provided the option to at least compare our results to adult studies. Moreover, given the mean age of the studied population the PSWQ seemed more appropriate than the PSWQ-Children [18] or studies that use instruments that are tapping into more general anxiety symptoms but not specifically into worry itself (e.g., the revised children’s anxiety and depression scale; [19]). Lastly, the present sample largely comprised adolescent girls with the restrictive subtype of AN, relative to the binge/purge subtype. Additionally, as this study was novel in investigating relations between IU, worry and weight and shape concerns in adolescent girls, it was decided to investigate the sample as a whole rather than in subtypes. However, future studies may focus on distinguishing subtypes of AN.

## Conclusions

In sum, this study contributes to the understanding of distinguishing components of IU and their associations with weight and shape concerns as it partly confirms our expectancy; inhibitory (but not prospective) IU co-occurs with weight and shape concerns in a large adolescent AN sample. This might indicate the importance of core cognitive anxiety-related processes such as IU in adolescent girls with AN. Furthermore, special attention could be given to the role of inhibitory IU within research as well as in interventions. A better understanding of inhibitory IU and weight and shape concerns might be helping in disentangling the complexity of body image disturbances, especially in adolescent girls when biologically driven changes of their bodies might lead an intolerable feeling of uncertainty. In contrast to our expectations, worry does not seem to be integrated in the cognitive processes of adolescent girls with AN as of yet. The precise nature and function of general worry seems to be more complex to interpret.

## Data Availability

Supporting data are unfortunately unavailable as a consequence of the strict regulations regarding the privacy of the patients.

## Abbreviations

AN: Anorexia nervosa
ANR: Anorexia nervosa restrictive subtype
ANBP: Anorexia nervosa binge/purge subtype
APA: American Psychiatric Association
DSM-5: Diagnostic and Statistical Manual of mental Disorders
ED: Eating disorders
EDE: Eating Disorder Examination
CBT: Cognitive behavioral therapy
IU: Intolerance of uncertainty
IUS-12: Intolerance of uncertainty scale
PSWQ: Penn State Worry Questionnaire
